# Natural history of ovarian endometrioma in pregnancy

**DOI:** 10.1186/1472-6874-14-128

**Published:** 2014-10-15

**Authors:** Katie Pateman, Francesca Moro, Dimitrios Mavrelos, Xulin Foo, Wee-Liak Hoo, Davor Jurkovic

**Affiliations:** Department of Obstetrics and Gynaecology, University College London Hospital, 235 Euston Road, London, NW1 2BU UK

**Keywords:** Endometrioma, Decidualisation, Ovarian cyst, Pregnancy

## Abstract

**Background:**

Ovarian endometriomas are classified as benign ovarian lesions. During pregnancy endometriomas may undergo major morphological changes which are referred to as ‘decidualisation’. Decidualised ovarian endometrioma may resemble malignant ovarian tumours on ultrasound examination. The aim was to study variations in the morphology and size of ovarian endometriomas diagnosed on ultrasound during pregnancy.

**Methods:**

We searched our database to identify pregnant women who were diagnosed with ovarian endometriomas on ultrasound in order to study the effect of pregnancy on their morphological characteristics. In women who underwent serial scans during pregnancy we examined the changes in the size of endometriomas with advancing gestation.

**Results:**

Twenty four patients with a total of 34 endometriomas were included in the analysis. All women were managed expectantly during pregnancy. On the first ultrasound scan 29/34 (85.3%, 95% CI 73.4 - 97.2) endometriomas appeared unilocular with fine internal echoes (‘ground glass’ contents) and they were poorly vascularised on Doppler examination. 1/34 (2.9% 95% CI 0.0 - 8.5) endometrioma was multilocular, with regular margins, ‘ground glass’ contents and it was also poorly vascularised. 4/34 (11.8%, 95% CI 1.0 - 22.6) had sonographic features suggestive of decidualisation such as thick and irregular inner wall, papillary projections and highly vascular on Doppler examination. The endometriomas showed a tendency to decrease in size during pregnancy.

**Conclusions:**

Pregnancy has a major effect on the size and morphological appearances of ovarian endometriomas. Rapid regression of decidualised endometriomas is a helpful feature which could be used to confirm their benign nature.

## Background

The number of women diagnosed with ovarian cysts during pregnancy has increased in recent years mainly due to the widespread use of ultrasound in the first trimester. It has been reported that adnexal cysts can be seen on ultrasound scan in 4.1% to 24.9% of pregnant women [1, 2]. Ovarian endometriomas account for 4-5% of ovarian cysts diagnosed in early pregnancy [3]. The appearances of endometriomas on ultrasound are variable, ranging from unilocular cysts with low level echoes (‘ground glass’ echogenicity) [4, 5] to solid looking tumours [4–6]. Despite the variability in their appearances endometriomas are relatively easy to classify correctly on ultrasound examination with the reported sensitivity and specificity being as high as 92% and 97% respectively [4, 5, 7–9]. Hormonal changes associated with the pregnancy may cause alterations in the sonographic appearances of endometriomas which are referred to as decidualisation. Decidualisation is the process of endometrial change caused by high progesterone levels which increases glandular epithelial secretion, accumulation of glycogen and stromal vascularity. These changes create conditions which facilitate implantation and development of early gestation. Formation of ectopic decidua (deciduosis) during pregnancy is a well-documented phenomenon that is caused by the effect of progesterone on ectopic endometrium, such as in foci of endometriosis [10]. Decidualised endometriomas may develop extensive intraluminal papillary projections with increased blood flow which are similar to malignant ovarian tumours [11–13].

The process of decidualisation of ovarian endometrioma during pregnancy is well documented. Although the ultrasound features of decidualisation can mimic ovarian cancer this appears to be a transient phenomenon. Decidualised endometrioma are usually surgically removed. We found only ten women with decidualised endometriomas who were managed expectantly during pregnancy, one had a first trimester miscarriage and so was not included in these results. Eight of them underwent ovarian cystectomy or oophorectomy at the time of Caesarean section. In all eight cases the diagnosis of benign decidualised ovarian endometrioma was confirmed on histopathology [14–19].

Decidualised ovarian endometrioma may resemble malignant ovarian tumours on ultrasound examination which can cause anxiety to women and lead to unnecessary and potentially harmful surgical interventions. There are several reports in the literature describing appearances of decidualised ovarian endometrioma prior to surgery [6, 10, 14, 15, 19–25]. Little is known, however, about the proportion of endometriomas that undergo decidualisation and their other changes during pregnancy. The aim of this article is to present an overview of the sonographic characteristics of ovarian endometriomas in pregnancy and provide more information about their natural history.

## Methods

**Figure 1:**
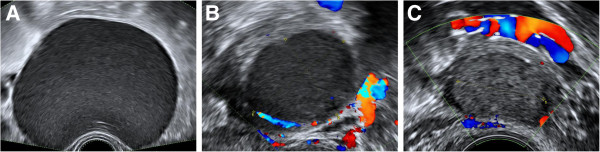
**The sonographic appearances of typical endometrioma.** Unilocular cysts with "ground glass" contents **[A]** and are poorly vascular or avascular on colour Doppler examination **[B and** **C]**.

We searched the database at the Early Pregnancy and Gynaecological Diagnostic and Outpatient Treatment Unit at University College London Hospital, London, UK to identify all pregnant women with a conclusive diagnosis of ovarian endometrioma. In all cases the diagnosis was confirmed by expert operators who had more than 10 years of experience in gynaecological ultrasound. The diagnosis was made using ‘pattern recognition technique’ which defined an endometrioma as a cyst typically located in the centre of the ovary with ‘ground glass’ echogenicity and surrounded by healthy ovarian tissue. On palpation the endometriomas were often adhered to the surrounding pelvic structures and on Doppler examination they were poorly vascularised [5] (Figure 1).

**Figure 2:**
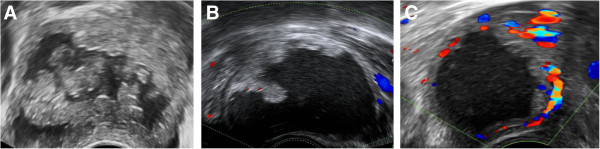
**Sonographic appearances of decidualised endometrioma.** Unilocular cysts containing hyperechoic fluid and irregular internal wall with prominent echogenic rounded papillary projections **[A]**. The papillary projections were typically highly vascular on Doppler examination **[B and C]**.

Decidualised endometrioma were diagnosed in women who presented with unilocular cysts containing hyperechoic fluid and irregular internal wall with prominent echogenic papillary projections [26]. The papillary projections were typically highly vascular on Doppler examination (Figure 2).

Maternal demographic data, indications for examinations, clinical symptoms and ultrasound findings were all recorded.

The site and morphology of ovarian endometriomas were recorded and the cysts were measured in three orthogonal planes. The surrounding ovarian tissue and ovarian capsule were not included in the measurements of the ovarian cyst. The volume of the cyst was calculated using the formula for an ellipsoid (4/3 Π abc, where a, b, and c are the semi-axes of the cyst). The side and morphology of the corpus luteum was also recorded.

The number of follow-up scans and the time intervals between scans were also recorded.

Ethical approval was sought from, and approved by, the Joint Research Office at University College London Hospital who deemed that full ethical approval was not required.

### Statistical analysis

The Kolmogorov-Smirnov test was used to test for normal distribution. Age, the time interval between ultrasound scans, ovarian cyst dimension and volume were not normally distributed and was expressed as median (interquartile range (IQR)). We used the Mann–Whitney *U*-test to compare the medians of non-normally distributed variables. Proportions were expressed as percentage (95% CI). We used the chi-square test and Fisher’s exact test to compare proportions.

## Results

We identified 24 pregnant women who were diagnosed with ovarian endometriomas in pregnancies between January 2009 and May 2013. The median maternal age was 35 years (IQR 32 – 37 years). 22/24 (92.0%, 95% CI 81.2 - 100) women were nulliparous and of these 11/22 (50.0%, 95% CI 29.1 - 70.9) were primigravid. Two remaining parous women were a primipara and a multipara.

20/24 (83.3%, 95% CI 68.4 - 98.2) women attended for their initial ultrasound scan in the first trimester of pregnancy, whilst the remaining 4/24 (16.7%, 95% CI 1.8 - 31.6) women had their first scans between 13 – 19 weeks’ gestation. The median gestational age at presentation was 9^+3^ weeks (IQR 6 – 12^+3^). Indications for ultrasound scans at presentation were: lower abdominal pain in 8/24 (33.3%, 95% CI 14.4 - 52.2), vaginal bleeding in 6/24 (25.0%, 95% CI 7.7 - 42.3), previous history of miscarriage in 5/24 (20.8%, 95% CI 4.6 - 37.0), and incidental finding of ovarian cyst on the routine 12 weeks’ scan in 5/24 (20.8%, 95% CI 4.6 - 37.0) women.

There were 34 cysts in total: 15/24 (62.5%, 95% CI 43.1 - 81.9) women had single unilateral endometrioma, 5/24 (20.8%, 95% CI 4.6 - 37.0) had multiple unilateral cysts and 4/24 (16.7%, 95% CI 1.8 - 31.6) had bilateral endometriomas. 9/24 (37.5%, 95% CI 18.1 - 56.9) women were surgically diagnosed with endometriosis prior to their pregnancies.

On the initial scan 29/34 (85.3%, 95% CI 73.4-97.2) cysts appeared unilocular with fine internal echoes ("ground glass" contents) and they were either avascular or poorly vascular on Doppler examination. 1/34 (2.9% 95% CI 0.0 - 8.5) cysts were multilocular and 4/34 (11.8%, 95% CI 1.0 - 22.6) cysts had sonographic features suggestive of decidualisation.

**Figure 3:**
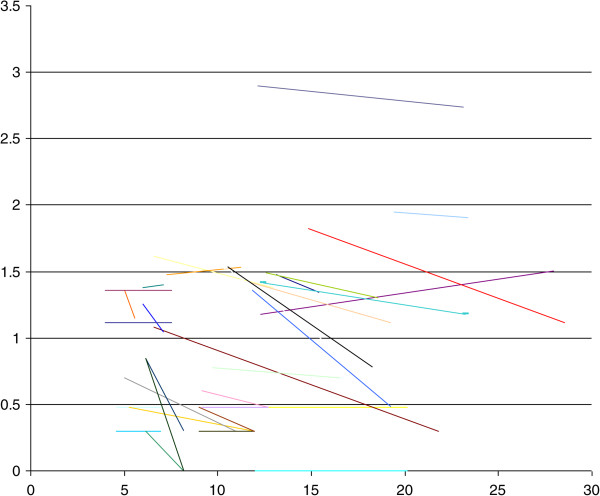
**Change in ovarian endometrioma volume over advancing gestation.** Y axis: Log of cyst volume (mls). X axis: Gestation (weeks).

In 15 women with unilateral cysts, the corpus luteum was present in the contralateral ovary in 11/15 (73.3%, 95% CI 50.9 - 95.7) cases and in the ipsilateral ovary in 4/15 (26.7%, 95% CI 4.3 - 49.1) cases.22/34 (64.7%, 95% CI 48.6 - 80.8) cysts decreased in size during pregnancy, 9/34 (26.5%, 95% CI 11.7 - 41.3) were unchanged during follow up and 3/34 (8.8%, 95 CI 0.0 - 18.3) cysts increased in size (Figure 3).

The median volume of the cysts at initial examination was 14.0 ml (IQR 3.0 - 30.0). The median rate of volume change of all endometriomas during the study period was -0.5 ml/week (IQR -2.8 to 0) or -5.2% per week (IQR -10.9 to 0).

There was no significant difference between decidualised and non-decidualised cysts in median volume at presentation (54.0 ml [IQR 23.5 to 77.0] vs. 12.5 ml [IQR 3.0 to 26.0], p = 0.105), absolute percent change (-29.1% [IQR -70.8 to -11.0] vs. -32.1% [-52.5 to 0.0], p = 0.661) rate of change (-4.2% per week [-6.3 to -2.3] vs. -5.2% per week [IQR -11.25 to 0.0], p = 0.897).

All women were managed expectantly during the pregnancy. One woman diagnosed with a decidualised endometrioma had ovarian cystectomy at the time of her Caesarean section and the histology report confirmed the pre-operative ultrasound diagnosis. 4/24 women who attended for postnatal scan had a reduction in the cysts volume compared to the initial measurements between 45.9% and 88.5%.

## Discussion

Our study has shown that pregnancy has a major effect on the biological behaviour of ovarian endometriomas. The majority of cysts decrease in size during pregnancy with the rate of regression being particularly high in decidualised endometriomas. This is in agreement with a widely held view that pregnancy has beneficial effect on endometriosis. High progesterone levels produced during pregnancy and temporary cessation of menstrual cycles and induce apoptosis are probably the main factors causing regression of endometriosis [27, 28].

We also found that 12% of endometrioma undergo decidualisation. Our findings are in agreement with a retrospective analysis reported by Ueda et al. [18] who also found signs of decidualisation in 12% of ovarian endometrioma during pregnancy. There are no published reports of decidualised endometriomas occurring outside of pregnancy for instance occurring in women receiving exogenous progesterones in form of pill, hormone implants or levonorgestrel-releasing intrauterine system. This could be explained by the high placental progesterone production which exceeds the amounts given during exogenous therapy. In addition, increased tissue perfusion and oedema caused by pregnancy could contribute to the formation of intraluminal mucosal folds and vascular papillary projections [29].

The reported prevalence of endometriomas in pregnancy is between 0.2% and 0.3% [2, 3]. Despite their relatively rarity they account for approximately a quarter of all surgical interventions for ovarian cysts detected during pregnancy [3].

A review of the literature showed that there is limited information regarding behaviour of endometriomas during pregnancy (Table 1). The first report of decidualised endometrioma in pregnancy was published by Miyakoshi in 1998 [10]. Since then, a number of further cases have been reported [6, 14–26, 30]. These reports provided descriptions of ultrasound features of decidualised endometriomas during pregnancy. In many cases decidualised endometriomas resembled ovarian malignancy. 28/31 (90%) women were offered surgical intervention, but two women opted for expectant management instead. In those who went ahead with the operation there were 9/26 (35%) laparotomies, 2/26 (8%) laparoscopies and the surgical approach was not described in the remaining fifteen cases. In 9/14 women with reported pregnancy outcomes following surgery there were 8/9 (89%) live births at term and one woman suffered a second trimester miscarriage. Although these findings were reassuring other studies have shown that surgical intervention in pregnancy may be associated with increased risks to maternal and fetal health [31]. A recent meta-analysis showed that risk of miscarriage is significantly higher in women undergoing laparoscopic compared to open appendicectomy in pregnancy [32]. In recent years laparoscopy has become the method of choice for excision of ovarian cysts in both non-pregnant and pregnant women. Although there is no specific data regarding the risks of laparoscopic cystectomy/oophorectomy in pregnancy, it is possible that they could be similar to laparoscopic appendicectomy. However, the risk of laparoscopic appendicectomy may be higher due to concomitant infection. In addition women with suspected ovarian cancer undergoing surgery are also likely to suffer psychological harm due to anxiety and stress [33].

**Table 1:** **The management of decidualised endometriomas in the literature**

Author	No of cases	Management	Surgical approach	Intervention	Gestation at intervention	Pregnancy outcome
Sammour	2	Surgical	Laparoscopy converted to laparotomy	Oophorectomy	16/40	NVD at term
		Surgical	Laparoscopy	Salpingo-oophorectomy	NS	NVD at term
Tazegul	1	Surgical	NS	Cystectomy	12/40	NS
Poder	1	Expectant			38/40	Caesarean section and salpingo-oophorectomy
Guerriero	1	Expectant			37/40	Caesarean section, frozen section, cystectomy
Fruscella	1	Surgical	Laparotomy	Cystectomy	18/40	Caesarean section at term
Miyakoshi	1	Surgical	Laparotomy	Salpingo-oophorectomy	22/40	NS
Tanaka	1	Surgical	NS	Cystectomy	12/40	NS
Barbieri	3	Surgical	Laparoscopy	Frozen section, cystectomy	14/40	NVD at term
		Expectant (declined surgery 24/40)			36/40	Concomitant Caesarean section and frozen section, cystectomy
		Expectant (declined surgery at 10/40)		Expectant	10/40	Miscarriage
Iwamoto	1	Surgical	Laparotomy	Salpingo-oophorectomy	22/40	NS delivery at term
Yoshida	2	Surgical	Laparotomy	Oophorectomy	28/40	NS delivery at term
		Surgical	Laparotomy	Cystectomy	16/40	NS
Sayasneh	1	Expectant		Expectant		NVD at term
Machida	3	Surgical	Laparotomy	Salpingo-oophorectomy	19/40	PROM - TOP 21/40
		Surgical	Laparotomy	Salpingo-oophorectomy	14/40	NVD at term
		Surgical	Laparotomy	Frozen section, cystectomy	14/40	NVD at term
Asch	1	Surgical	NS	NS	NS	NS
Ueda	3	Surgical	NS	Salpingo-oophorectomy	NS	NS
		Surgical	NS	Salpingo-oophorectomy	NS	NS
		Expectant				NS
Mascilini	13^§^	Surgical	NS	NS	NS	NS
	5	Expectant				Concomitant Caesarean section and NS cystectomy or oophorectomy

NS not specified, NVD normal vaginal delivery, PPROM premature rupture of membranes, TOP termination of pregnancy.

^§^The case by Fruscella et al. is also included in this total.

Our data show that expectant management of decidualised endometrioma is likely to be safe with a fast regression rate. We have also found that decidualised endometrioma tend to regress particularly fast. This is a reassuring feature which may be used to help to avoid unnecessary interventions due to difficulties in differential diagnosis between decidualised ovarian endometrioma and ovarian cancer on the initial scan. Our findings are also in agreement with a recent published study by Mascilini et al. [19], who also noted that the highly vascularised papillary projections are more rounded with a smooth contour.

Decidualised endometriomas tend to resemble borderline ovarian tumours which also contain multiple papillary projections and tend to be surrounded by normal ovarian parenchyma. Borderline tumours typically increase in size during follow up, whilst decidualised endometrioma show the opposite trend. Other ultrasonographic features of endometriosis, such as obliteration of the pouch of Douglas and deep infiltrating endometriotic nodules [34] may also help facilitate the difference between borderline ovarian tumours and decidualised endometriomas. Additional tests which are used to differentiate between benign and malignant cysts such as serum CA125 are not helpful in pregnancy. Serum CA125 levels in particular are significantly higher in pregnant women which may lead to a false positive diagnosis of ovarian cancer during pregnancy [35]. Other complications such as ovarian torsion are unlikely to occur in ovarian endometriomas which tend to be firmly adhered to the surrounding pelvic structures.

The limitation of our study was its retrospective nature with no structured follow-up. The majority of patients did not attend for a post-natal scan and therefore the changes following delivery could not be assessed. In addition, the proportion of decidualised cysts may have been overestimated as women with small non-decidualised endometriomas are less likely to be referred for specialised gynaecology examination. The majority of women were managed conservatively and histological confirmation of ultrasound diagnosis is lacking in most cases.

Future prospective studies should provide more information about the natural history of endometriomas in pregnancy and pregnancy outcomes following surgical interventions. More information is also needed regarding recurrence rates following uncomplicated pregnancy and delivery.

## Conclusions

Pregnancy has a major effect on the size and morphological appearances of ovarian endometriomas. The decidualisation rate of ovarian endometriomas diagnosed in pregnancy in our cohort was 12%. Rapid regression of decidualised endometriomas is a helpful feature which could be used to confirm their benign nature.

## Authors’ information

Gynaecology Diagnostic and Outpatient Treatment Unit

Lower ground floor, EGA Wing, University College London Hospital

235 Euston Road, London, NW1 2BU, UK.

## Abbreviations

CA: 125 Cancer antigen 125
CI: Confidence intervals
IQR: Interquartile range
ML: Millilitres
NS: Not specified
NVD: Normal vaginal delivery
PROM: Premature rupture of membranes
TOP: Termination of pregnancy.
